# The Bacterial Protein Azurin Impairs Invasion and FAK/Src Signaling in P-Cadherin-Overexpressing Breast Cancer Cell Models

**DOI:** 10.1371/journal.pone.0069023

**Published:** 2013-07-19

**Authors:** Nuno Bernardes, Ana Sofia Ribeiro, Sofia Abreu, Bruna Mota, Rute G. Matos, Cecilia M. Arraiano, Raquel Seruca, Joana Paredes, Arsenio M. Fialho

**Affiliations:** 1 Institute for Biotechnology and Bioengineering, Center for Biological and Chemical Engineering, Instituto Superior Técnico, Lisbon, Portugal; 2 Institute of Molecular Pathology and Immunology of the University of Porto (IPATIMUP), Porto, Portugal; 3 Instituto de Tecnologia Quimica e Biológica (ITQB), Universidade Nova de Lisboa, Av^a^ da República, Oeiras, Portugal; Vanderbilt University, United States of America

## Abstract

P-cadherin overexpression occurs in about 30% of all breast carcinomas, being a poor prognostic factor for breast cancer patients. In a cellular background of wild-type E-cadherin, we have previously shown that its expression promotes invasion, motility and migration of breast cancer cells due to the induced secretion of metalloproteases (MMPs) to the extracellular medium and to the concomitant shedding of a pro-invasive soluble form of this protein (sP-cad). Azurin is secreted by *Pseudomonas aeruginosa* and induces *in vitro* and *in vivo* cytotoxicity after its preferential penetration in human cancer cells relative to normal cells. Three different breast cancer cell lines, MCF-7/AZ.Mock, MCF-7/AZ.Pcad and SUM149 were treated with sub-killing doses of azurin. Invasion of these cells was measured using Matrigel Invasion Assays and MTT assays were performed to determine cell viability upon treatment and the effects on cadherins expression was determined by Western blot and Immunofluorescence. Gelatin Zymography was used to determine activity of MMP2 in the conditioned media of azurin treated and untreated cells and the phosphorylation levels of intracellular signaling proteins were determined by Western blot. The invasive phenotype of these breast cancer cells was significantly reduced by azurin. Azurin (50–100 µM) also caused a specific decrease on P-cadherin protein levels from 30–50% in MCF-7/AZ.Pcad and SUM149 breast cancer cell lines, but the levels of E-cadherin remain unaltered. More, the levels of sP-cad and the activity of MMP2 were reduced in the extracellular media of azurin treated cells and we also observed a decrease in the phosphorylation levels of both FAK and Src proteins. Our data show that azurin specifically targets P-cadherin, not E-cadherin, abrogating P-cadherin-mediated invasive effects and signaling. Therefore, azurin could possibly be considered a therapeutic tool to treat poor-prognosis breast carcinomas overexpressing P-cadherin in a wild type E-cadherin context.

## Introduction

Cell invasion is a critical step in cancer progression [1]. Invasive cancer cells have significant altered properties, namely in polarity and morphology, as well as in their ability to adhere to other cells and to the extracellular matrix (ECM) components. Indeed, cell-cell adhesion and cell-ECM adhesion need to be very tightly regulated for the maintenance of a normal epithelial architecture [2].

Classical type I cadherins, namely E-cadherin (or epithelial-cadherin - Ecad), are crucial players that regulate cell-cell adhesion. During epithelial-to-mesenchymal transition (EMT), E-cadherin is usually down-regulated or functionally inactivated and *de novo* expression of other cadherins is frequently observed, a process named cadherin switch. These de-regulations cause alterations that are reflected in terms of intracellular signaling, as well as in cell behavior, as loss of cell polarity and acquisition of invasive capacity [3].

P-cadherin (Pcad) overexpression occurs in 30% of invasive breast carcinomas, being associated with poor patient prognosis. Interestingly, in some metastatic breast cancer models, as well as in high-grade primary carcinomas and in the aggressive local inflammatory breast cancer, E-cadherin expression is maintained alongside with aberrant expression of P-cadherin [4], [5]. We have previously found that the increased expression of P-cadherin promotes invasive effects in breast cancer cells, which can be, at least in part, attributed to the release of a soluble form of P-cadherin (sP-cad) to the extracellular media, that is capable by itself to cause invasion of E-cadherin positive, non-invasive, cell lines [6]. Also, increased expression and activity of matrix metalloproteases (MMPs), namely MMP-1 and MMP-2, are involved in cell invasion mediated by P-cadherin overexpression [6], as well as the activation of the intracellular non-receptor tyrosine kinases FAK and Src, that regulate a wide number of signaling pathways involved in cell spreading, adhesion, migration, invasion, survival, proliferation, differentiation and angiogenesis [7].

Azurin is a small copper protein (14 kDa), water-soluble, produced by the bacterium *Pseudomonas aeruginosa*. Besides its described function as a redox partner in electron transfer reactions, azurin can act as an anticancer agent exerting cytotoxicity *in vitro* against several cancer derived cell lines and promotes tumor regression in xenografted mice [8]–[11]. In cultured cells, azurin enters cancer cells preferentially when compared to normal cells derived from the same tissue, mediated by a portion of the protein spanning residues 50–77 (termed p28), which adopts an amphipathic alpha-helical conformation [12]. p28, as a lead compound supported by CDG Therapeutics, has finished Phase I clinical trial, which defined it as an anticancer agent under an investigational new drug application (IND 77,754) approved by the Food and Drug Administration.

In mechanistic terms, it is known that azurin targets different cell proliferation pathways. Once internalized, azurin can interact directly with p53 and stabilize it, increasing its protein levels in both nuclear and cytoplasmic fractions [10], [12]–[15]. Besides p53-mediated cell cytotoxicity, this protein also binds the extracellular EphB2 tyrosine kinase receptor, being able to prevent the tumor progression caused by the binding of the natural ligand ephrinB2 [16]. Recently, p28 was found to penetrate endothelial cells (HUVEC) and mediate the decrease in their motility and migration, which was associated with an inhibition of VEGFR-2 (vascular endothelial growth factor receptor 2) kinase activity [17]. Moreover, the levels of phosphorylated FAK (Focal adhesion kinase) and Akt proteins were also reduced, altering the intracellular architecture of endothelial cytoskeleton and cell contact proteins that limit endothelial cell motility and migration.

Based on the previous described anticancer and anti-migratory effects of azurin, we hypothesized that it could also be used as a therapeutic tool in highly aggressive breast cancers overexpressing P-cadherin, being able to inhibit its pro-invasive effects. In this work, we describe, for the first time, that azurin decreases P-cadherin expression at the cellular membrane and inhibits P-cadherin-induced breast cancer cell invasion at sub-killing doses. Furthermore, we identified that this phenomenon is associated to a MMP-2 decreased activity in the extracellular media and decrease in FAK/Src complex signaling, possibly mediating the anti-invasive effects of azurin in P-cadherin-overexpressing breast cancer cells.

## Materials and Methods

### Antibodies

Primary antibodies: P-cadherin (Western blot: clone 56, BD Biosciences, Lexington, KY, USA; immunofluorescence: Cell Signaling Technology, Boston, MA, USA), E-cadherin (clone HECD1, Takara Bio Inc., Shiga, Japan), β-actin (I-19, Santa Cruz Biotechnologies, CA, USA), total FAK (BD Biosciences), pFAK Y397(Cell Signaling), total Src (Cell Signaling), pSrc Y416 (Cell Signaling), total Akt (Santa Cruz Biotechnologies), pAkt S473 (Cell Signaling).

### Bacteria and Growth Media

Azurin-encoding gene from *P.aeruginosa* PAO1 was amplified by PCR, using genomic DNA as a template. Forward and reverse primers used were: 5′-CGGGATCCGCCGAGTGCTCGGTGGACAT-3′ and 5′-CCCAAGCTTGCATCACTTCAGGGTCAGGGT-3′. Azurin gene was placed downstream the T7 promoter in the pWH844 vector. *E.coli* SURE strain was used as host for expression of the protein in the following conditions: cells were incubated overnight in LB medium at 37°C with 150 µg/ml of ampicillin and cultured, at an initial optical density at 640 nm (OD_640_) of 0.1, in SB medium (3.2% tryptone, 2% yeast extract and 0.5% NaCl). At OD_640_ of 0.65, IPTG was added to the culture at a final concentration of 0.2 mM and grown for 4 h at the same conditions. Cells were harvested by centrifugation at 8000 rpm for 10 minutes at 4°C, washed once and resuspended in buffer I (10 mM Imidazol, 0.2 M mM sodium phosphate, 0.5 M NaCl, pH 7.4). Cells were stored at −80°C for further use.

### Protein Purification

Cells were disrupted by sonication and the purification steps were performed by histidine affinity chromatography, using HisTrapTM HP columns (GE Healthcare). Briefly, disrupted cells were centrifuged for 5 min, at 17600×g and 4°C; the supernatant was centrifuged again at the same conditions for 1 h. The clarified extract was then loaded into a 5 ml HisTrap HP column equilibrated with START buffer (10 mM Imidazol, phosphate buffer: 0.2 M sodium phosphate, 1 M NaCl, pH 7.4). Protein elution was achieved with a continuous imidazole gradient (from 20 to 500 mM) in the same buffer. After purification, protein was immediately de-salted and buffer exchanged to phosphate-buffered saline (PBS) pH 7.4, in a HiPrep 26/10 Desalting column (GE Healthcare) in an AKTA purifier system, following the manufacturer’s instructions. Finally, protein was concentrated by centrifugation at 4°C with Amicon Ultra Centrifugal Devices (Milipore), with a molecular mass cutoff of 10 kDa. Purified protein was passed through 1 ml Detoxi-GelTM Endotoxin Removing column (Thermo Scientific) to remove endotoxins from *E. coli* host strain. At all steps, protein concentration was assessed with BCA™ Protein Assay kit (Thermo Scientific), following the manufacturer’s instructions. The purity of protein was analyzed by sodium dodecyl-polyacrylamide gel electrophoresis (SDS-PAGE).

### Cell Culture

Two distinct human breast cancer cell lines were used in this study: MCF7/AZ [kindly provided by Prof. Marc Mareel (Ghent University, Belgium); MCF-7/AZ.Mock and MCF-7/AZ.Pcad [18] were stably transduced with empty vector or *CDH3*/Pcadherin cDNA, respectively] and SUM149 [19] [kindly provided by Prof. Stephen Ethier (University of Michigan, MI, USA]. Both cell lines were routinely maintained at 37°C, 5% CO_2_, in the following media (Invitrogen Ltd, Paisley, UK): 50% DMEM and 50% HamF12, supplemented with 10% heat-inactivated fetal bovine serum (Lonza, Basel, Switzerland), 100 IU/ml penicillin and 100 mg/ml streptomycin (Invitrogen). SUM149 medium was additionally supplemented with 5 µg/ml of insulin and 1 µg/ml of hydrocortisone (Sigma-Aldrich, St. Louis, MO, USA).

MCF-7/AZ cell line was retrovirally stable transduced to encode human P-cadherin cDNA (MCF-7/AZ.Pcad cell line), as previously described (Paredes et al. 2004). MCF-7/AZ.Mock cell line, encoding only EGFP, was used as a control. SUM149 cell line constitutively expresses high levels of P-cadherin. All the cell lines used express normal levels of E-cadherin.

### MTT Cell Viability Assay

MTT [3-(4,5 dimethylthiazol-2-yl-2,5 tetrazolium bromide)] assays were used to determine viability of breast cancer cells upon azurin exposure. Breast cancer cells were seeded in 96-well plates (3 replicates) (Orange Scientific) at a density of 2×10^4^ cells (MCF-7/AZ cell lines) or 5×10^4^ cells (SUM149). After 24 h, medium was exchanged and fresh azurin or a similar volume of media without protein (100 µL) were added. After another 24 h (SUM149) or 48 h (MCF-7/AZ cell lines), 10 µL of MTT (5 mg/ml) were added to each well and incubated at 37°C for 3.5 h. Reaction was stopped with the addition of 40 mM HCL in isopropanol. MTT formazan formed was spectrophotometrically read at 590 nm in a 96-well plate reader. Untreated cells were used as control, in order to to determine the relative cell viability of treated cells.

### Protein Extraction and Western Blot Analysis

Cultured cells, treated with azurin (50 or 100 µM) or untreated, were lysed using catenin lysis buffer (1% Triton X-100, and 1% NP-40 in deionized PBS), supplemented with 1∶7 protease inhibitor cocktail (Roche Diagnostics GmbH, Germany) and with 1∶100 of phosphatase inhibitor cocktail 3 (Sigma). Cells were washed twice with PBS and allowed to lyse in 100 µL of catenin lysis buffer for 10 minutes, at 4°C. Lysed cells were collected and vortexed three times, for 10 seconds, prior to centrifugation at 17600×g for 10 minutes at 4°C. Total protein quantification was performed with BCA™ Protein Assay kit (Pierce). 20 (E- and P-cadherin, total FAK, Src and Akt) or 30 µg (phosphorylated FAK, Src and Akt) of the total protein lysate was dissolved in sample buffer [Laemmli with 5% (v/v) 2-β-mercaptoethanol and 5% (v/v) bromophenol blue], boiled for 5 minutes at 95°C, and separated by SDS-PAGE. Proteins were transferred onto nitrocellulose membranes (Pall) at 120 V for 1 hour and 30 minutes. Membranes were blocked with 5% (w/v) non-fat dry milk in PBS containing 0.5% (v/v) Tween-20 (PBS-T) or 5% BSA (for phosphorylated protein detection) for 1 hour, incubated with primary antibodies, overnight at 4°C (E-cadherin, P-cadherin and total FAK,Src and Akt) or for 1.5 h at room temperature in 5% BSA (w/v) (pFAK, pSrc and pAkt), and washed three times for 5 minutes with PBS-T. Membranes were then incubated for 1 hour with secondary antibodies, conjugated with horseradish peroxidase. Proteins were detected through the addition of ECL reagent (Pierce) as a substrate and exposed to an autoradiographic film (Amersham). Three experiments were independently performed and representative results are shown. Signal quantifications were performed using ImageJ and results are presented as the ratio between the signal intensities in azurin treated samples to untreated cells, both normalized to the respective actin band intensities.

### Immunofluorescence

Cells were cultured on glass coverslips and treated with azurin at different concentrations. After the desired exposition time, medium was collected and cells were washed twice with PBS. Fixation was performed with NH_4_Cl for 20 minutes at room temperature. Permeabilization was achieved with 1% Triton X-100 in PBS for 5 minutes at room temperature and coverslips were blocked with 5% BSA solution in PBS for 30 minutes. Primary antibodies were added for 1 hour at room temperature as follows: 1∶100 dilution for E-cadherin antibody and 1∶50 dilution for P-cadherin antibody. After the incubation time, cells were washed three times for 5 minutes with PBS and incubated with secondary antibodies for 1 hour, at room temperature, at 1∶500 dilution as described: mouse polyclonal conjugated with ALexa-488 for E-cadherin and rabbit polyclonal antibody conjugated with Alexa-594 for P-cadherin. Each sample was washed with PBS after the incubation period and mounted with Vectashield (Vector Laboratories Inc., Burlingame, CA, USA) containing 4,6-diamidine-2-phenylindolendihydrochloride (DAPI). Cell staining was observed in a Zeiss microscope (Imager Z1) and images acquired using the Axiovision software.

### Quantitative Real-Time PCR

Total RNA from MCF-7/AZ and SUM149 cell lines was extracted using the RNeasy Extraction kit (Qiagen), according to the manufacturer’s instructions. Samples were subjected to treatment with DNase (Qiagen) during the extraction procedure. qRT-PCR was performed using gene-specific TaqMan probes (Applied Biosystems, Foster City, CA). Analysis was performed using the ABI PRISM 7500 Sequence Detection System Instrument and software (Applied Biosystems). *CDH1* and *CDH3* relative quantifications between treated and untreated samples were determined by the ΔΔCt method using the internal standard human *18S* to normalize cDNA quantity.

### Matrigel Invasion Assays

Matrigel Invasion assay was performed according to the manufactureŕs instructions (BD Coat Matrigel Invasion Chambers, BD Biosciences). Briefly, Matrigel inserts containing an 8 micron pore size PET membrane with a thin layer of Matrigel Basement Membrane Matrix were pre-incubated with serum-free media for 2 hours at 37°C. 5×10^4^ cells for MCF7/AZ.Mock and MCF7/AZ. Pcad, and 2.5×10^4^ cells for SUM149, were seeded in the upper compartment with or without azurin (control). After 48 h or 24 h, invasive cells were colored with DAPI and counted under the microscope. In each condition, 10 independent fields were counted and the average of these fields considered as the mean number of invasive cells per condition.Results are presented as the fold change in invasion of cells in comparison with the MCF-7/AZ.Mock untreated cells.

### Gelatin Zymography

Cell conditioned media was collected to analyse the activity of MMP2, using gelatin zymography. Cells were cultured in 6-well plates coated with collagen type I (1 mg/ml). Gelatin gels were loaded with a 15 µL sample for each condition, mixed with sample buffer [0.03% bromophenol blue, 0.25 M Tris-HCl pH 6.8, 10% SDS (w/v) and 4% sucrose (w/v)]. Electrophoresis was performed on 10% polyacrylamide gels containing 0.1% (w/v) of gelatin (Sigma) at 80V, under non-reducing conditions. Gels were washed twice in 2% (v/v) Triton X-100 (Sigma), 30 minutes each time, at room temperature, to remove SDS. After, gels were incubated in a Substrate Reaction Buffer (50 mM Tris-HCl, 5 mM CaCl_2_, pH 7.5, 1% Triton X-100) for 17 hours. After incubation, gels were stained in Coomassie Blue Staining Solution [0.1% (w/v) Coomassie Blue R250 in 10% acetic acid solution and 40% (v/v) methanol], for 30 minutes, and de-stained in 20% (v/v) methanol and 10% (v/v) acetic acid solution, until white bands against the blue background of the gels appeared. MMP2 was identified according to their molecular weight.

### Statistical Analysis

Data are expressed as mean values of a minimum of three independent experiments ± s.d. Student’s t-tests were performed to determine statistically significant differences. A *P* value <0.05 was considered statistically significant.

## Results

### Azurin Impairs Invasion of MCF-7/AZ.Pcad and SUM149 Invasive Breast Cancer Cells

**Figure 1:**
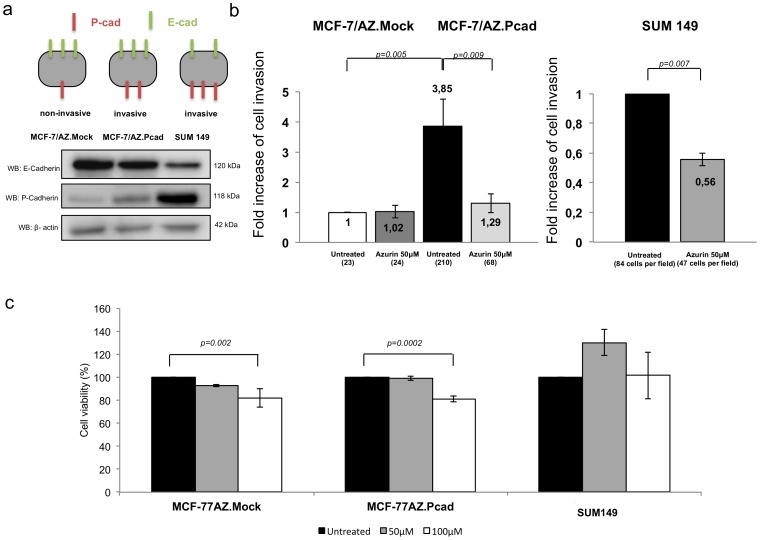
Azurin impairs invasion of breast cancer cells over-expressing P-cadherin. a) Schematic representation of the invasive profile of cell lines used in this work (upper panel) and E- and P-cadherin protein expression levels for each cell line (lower panel) b) Azurin decreases invasion of MCF-7/AZ.Pcad and SUM149 cells. Matrigel Invasion Assays showed that one single treatment of azurin at 50 µM for 48 h (MCF-7/AZ.Mock and MCF-7/AZ.Pcad) or 24 h (SUM149) significantly reduced the invasive behaviour of breast cancer cells.MCF-7/AZ.Mock cell line was used as a control and the invasion of this cell line was used to normalize the levels of invasion. c) Cell viability assessed with MTT assay of MCF-7/AZ.Mock, MCF-7/AZ.Pcad and SUM149 cell lines in the presence of azurin. Cells were plated in 96-well plates in the presence of 50 and 100 µM of azurin for 24 h (SUM149) or 48 h (MCF-7/AZ.Mock and MCF-7/AZ.Pcad) to match the time course of invasion assays for each cell line. Control cells received complete media without azurin.

Previous work from our group has demonstrated that invasion of breast cancer cells can be induced by P-cadherin overexpression in a wild-type E-cadherin context [6]. In fact, we showed that expression of P-cadherin in MCF-7/AZ cells induces an increase in cell invasion [6], whereas the knocking-down of P-cadherin by siRNA causes a decrease in the invasive behavior of SUM149 breast cancer cells [21]. Thus, in order to study if azurin could impair the invasion mediated by P-cadherin, these same human breast cancer cell models were used (Figure 1a). Using Matrigel Invasion Assays, we observed that a sub-killing dose of azurin (one single addition at 50 µM) reduces invasion of both MCF-7/AZ.Pcad and SUM149 breast cancer cells lines, 66% and 44%, respectively (Figure 1b). MCF-7/AZ.Mock cells did not change its non-invasive behavior after azurin treatment (Figure 1b).

The reduced invasion observed was not due to decreased cell viability, as assessed by MTT assays performed for the same azurin concentrations and exposure times (Figure 1c).

### Azurin Causes a Specific Decrease of P-cadherin Protein Levels in Human Breast Cancer Cells

**Figure 2:**
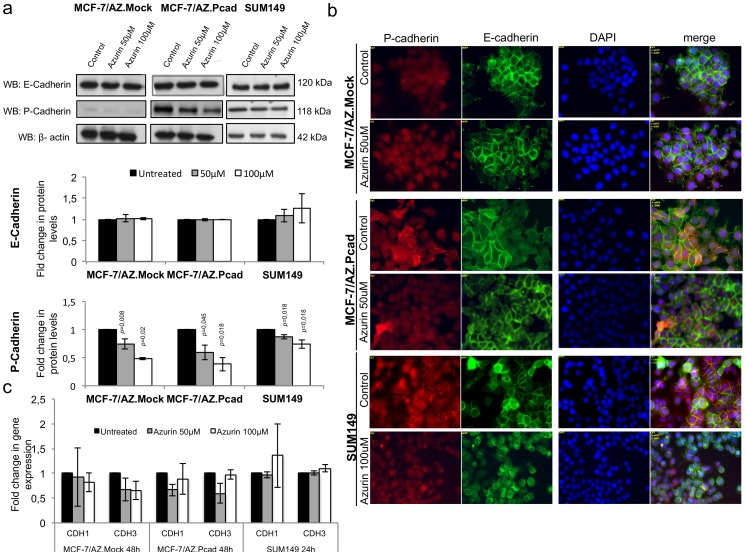
Azurin decreases P-cadherin protein levels. a) One single treatment with azurin at 50 µM and 100 µM for 48 h (MCF-7/AZ.Pcad) and 24 h (SUM149) (to match the time course of invasion assays) reduces the P-cadherin expression by Western Blot with no significant alteration at E-cadherin levels. Results are presented as the ratio of band intensity of target protein between azurin treated samples and control samples, both normalized to their respective actin band intensity b) Same results were observed by immunofluorescence analysis of both cadherins in the same treatment conditions. c) Azurin does not significantly change the expression levels of *CDH1*/E-cadherin or *CDH3*/P-cadherin at mRNA levels, as observed by qRT-PCR at the same conditions as for Westerm Blot analysis.

After observing the effects caused by azurin in the inhibition of invasion in P-cadherin overexpressing breast cancer cell lines, we decided to study the expression levels of both E- and P-cadherin after azurin treatment. MCF-7/AZ.Mock and MCF/AZ.Pcad were treated with 50 µM and 100 µM for 48 h, and presented a decrease in P-cadherin protein levels, while E-cadherin levels were not altered. P-cadherin was 30–50% reduced with one single dose of azurin. In the SUM149 cells, the same effects were observed, although less pronounced than for MCF-7/AZ cells, causing a decrease in P-cadherin levels of about 15–30% for one single treatment during 24 h (Figure 2a). In this cell line, it was also seen that E-cadherin levels were not significantly altered after azurin treatments.

Accordingly, the same results were found by immunofluorescence analysis (Figure 2b). Azurin treatments reduced the membrane levels of P-cadherin, whereas E-cadherin expression remained unaltered, with normal membrane cell localization. Despite the alterations observed in P-cadherin protein levels and cell localization, we could not observe significant differences at *CDH1*/E-cadherin and *CDH3*/P-cadherin gene expression levels (Figure 2c).

### Azurin Decreases Activity of MMP2 and the Soluble P-cadherin Levels in the Extracellular Media of P-cadherin Overexpressing Breast Cancer Cells

**Figure 3:**
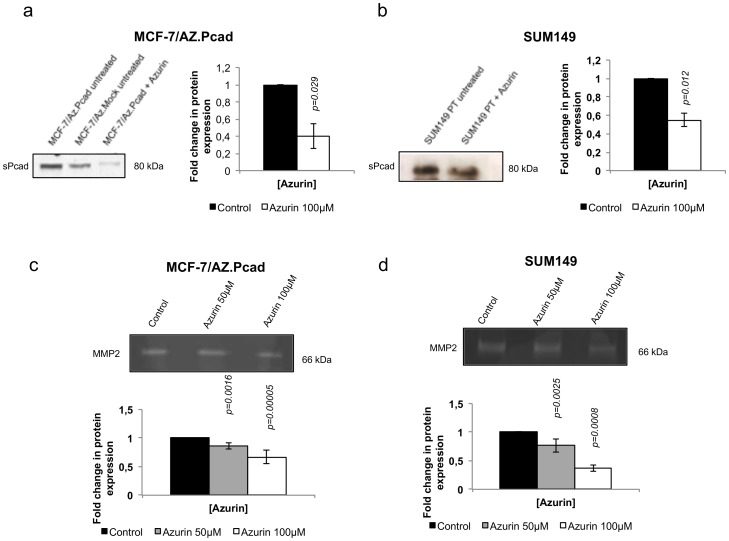
Azurin induced a decrease sPcad levels and MMP2 activity. Treatments with azurin (100 µM) leads to decreased levels of the pro-invasive soluble form P- Cadherin in the conditioned media of cultured cells, as observed by Western Blot, for both MCF-7/AZ.Pcad (a) and SUM149 (b). Gelatin zymography allowed the identification of MMP2 activity in the conditioned media of MCF-7/AZ.Pcad (c) and SUM149 (d) cells grown in a collagen type I matrix.

We have previously demonstrated that P-cadherin-induced invasion is mediated by the secretion of metalloproteases (MMP1/2) to the extracellular media, which will cleave the full-length P-cadherin, generating a soluble fragment of this protein (sP-cad) with pro-invasive activity [6]. Thus, we decided to assess the levels of sP-cad in the conditioned media of azurin treated cells. Accordingly with the decrease in P-cadherin levels at the cellular membrane, we also observed a decrease in the sP-cad levels into the extracellular media of MCF-7/AZ.Pcad and SUM149 cells (Figure 3a and 3b). We also investigated, after azurin treatment, the activity and expression of MMP2 associated with P-cadherin expression, using zymography of the conditioned media of MCF-7/AZ.Pcad and SUM149 cells. Our results show evidences of a decrease in the activity of this protease in both cell lines (Figure 3c and 3d), concomitant with the P-cadherin decrease.

### Azurin Decreases the Activity of FAK and Src Induced by P-cadherin Expression

Recently, it has been described that azurin can mediate the decrease in motility and migration of endothelial cells, by inhibiting VEGFR-2 kinase activity, inducing the decrease of the levels of phosphorylated FAK and Akt proteins [17]. Moreover, increased expression and activity of MMPs are involved in the activation of the intracellular non-receptor tyrosine kinases FAK and Src, regulating a wide number of signaling pathways, including cell spreading, adhesion, migration, and invasion [7],

Thus, since azurin is able to impair P-cadherin-induced cell invasion, as well as decreases MMP2 activity, we decided to explore if FAK and Src kinase activity could be impaired in a P-cadherin overexpression context.

**Figure 4:**
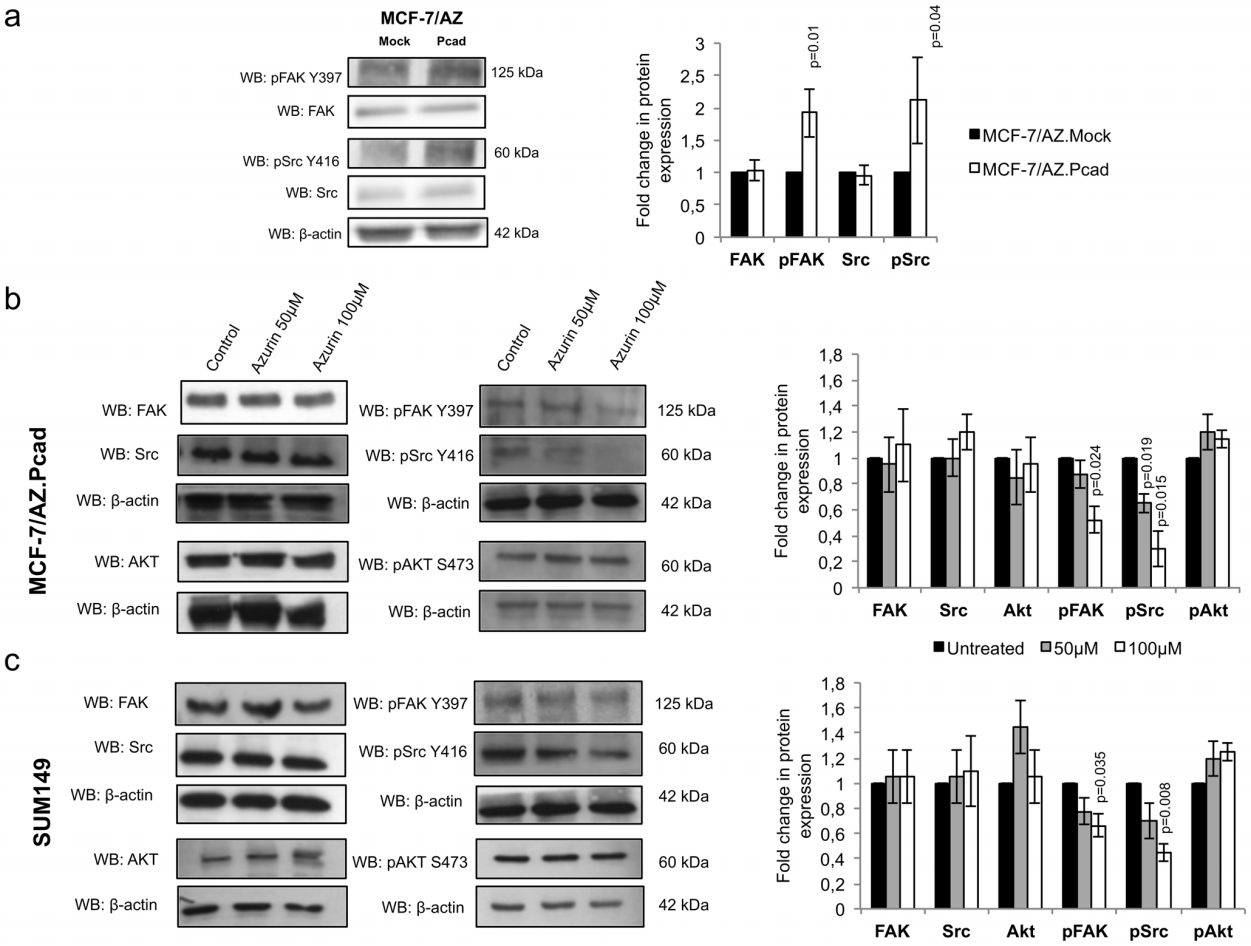
Effect of azurin in FAK-Src signaling. Azurin at 50 µM and 100 µM decreased phosphorylation levels of FAK Y397 and Src Y416 in both MCF-7/AZ.Pcad and SUM149 breast cancer cells, but not Akt S473, in a dose-dependent manner. Levels for total FAK, Src and Akt were also analyzed. Results are presented as the ratio of band intensity of target protein between azurin treated samples and control samples, both normalized to their respective actin band intensity.

In fact, overexpression of P-cadherin leads to an increase in the levels of phospho-FAK and phospho-Src proteins, with no significant alterations in the levels of total proteins (Figure 4a). Interestingly, we were able to see a decrease in the phosphorylation levels of both FAK Y397 and Src Y416 in a dose-dependent manner after azurin treatment in both cell models (Figure 4b and 4c). In contrast, no differences were observed for the total levels of these proteins between control and treated cells. We also analyzed the phosphorylation levels of Akt S473, to understand if the inhibitory effect mediated by azurin could be following that signaling pathway, but no differences were observed in this protein (Figure 4b and 4c).

## Discussion

P-cadherin expression in breast carcinomas is a marker for a subset of cancer with poor patient survival, particularly with the concomitant expression of E-cadherin. Recently, our group has showed that P-cadherin expression interferes with the normal invasive suppressive function of E-cadherin, being responsible for the highly aggressive phenotype of breast cancer cells within these tumors [6]. Since, so far, there is no targeted therapy to this subset of tumors, we decided to study, for the first time, if azurin could have an inhibitory action on P-cadherin overexpressing breast cancer cells. Azurin and its derived peptide p28 have been previously analyzed as anti-tumoral agents, inhibiting cancer cell growth, mainly by interfering with p53 protein [9], [10], [13], [14].

In order to achieve this goal, three different breast cancer cell lines expressing different levels of P-cadherin and with different p53 status were used: MCF-7/AZ.Mock and MCF-7/AZ.Pcad, which present a p53 wild type function, and SUM149, which constitutively overexpresses P-cadherin and shows a mutant form of p53. Using Matrigel Invasion Assays, we showed that azurin reduces invasion in P-cadherin-overexpressing cells, an effect that cannot be associated to a significant decreased viability of the cells.

Curiously, the effects on cell invasion seem to be related with a specific decrease in P-cadherin protein in the three cell lines analysed after azurin treatment, with no effects on E-cadherin levels. In that way, we cannot relate this specific effect with the described effects of azurin regarding its binding and modulation of p53 pro-apoptotic activity.This specificity shown for azurin effect on cadherins is very interesting, since P-cadherin expression is correlated to increased cell motility, cell migration and invasion [6], only in cell systems that are also positive for E-cadherin expression; in highly invasive melanoma, which lacks the expression of E-cadherin, P-cadherin expression is able to induce cell-cell contacts and decrease invasion [5], [20], [21]. Recently, the role of P-cadherin promoting invasion in E-cadherin wild type context was further elucidated by showing that P-cadherin promotes a disruption in the interaction of E-cadherin and cytoplasmic catenins [22]. Moreover, the expression of both these cadherins correlated significantly with high grade breast carcinomas and poor patient survival. *In vivo*, a breast cancer cell model expressing both cadherins was found more aggressive, with higher tumour growth when compared with the same model expressing only one of the cadherins by suppressing each cadherin by siRNA technology [22]. These results reinforce the anti-invasive role of azurin in this context, since its action was preferential to P-cadherin and not for E-cadherin. We could not observe a clear reduction in *CDH3*/P-cadherin gene expression, suggesting that other mechanisms at protein degradation level are mediating P-cadherin decrease. These mechanisms are now under investigation.

A known mechanism that determines at least part of the aggressiveness of P-cadherin expression is the release of soluble forms of this protein, sP-cad, to the extracellular media of the cells. This form is *per se* capable of causing cell invasion of non-invasive cells [6]. In the presence of azurin, and probably due to the caused P-cadherin decrease, lower levels of sP-cad are detected in azurin treated cells. This result is interesting, since soluble forms of classical cadherins have been associated with malignant effects [23]: soluble E-cadherin was associated with increased invasion and with the inhibition of normal E-cadherin-dependent cell-cell aggregation [24], and higher levels of soluble P-cadherin were found in nipple aspirate fluids of breast cancer patients than in healthy women [25].

The cleavage and shedding of P-cadherin, and therefore the higher invasive capacity of these cell lines, is mediated by MMPs. In fact, several tumorigenic processes are mediated by these proteases, namely the breakdown of extracellular components, which accounts greatly to the ability of tumor cells to invade the surrounding tissues through an extensive matrix remodeling [7]. MMPs also promote the release of bioactive molecules able to induce invasion, like the cleavage of laminin-5 γ2 chains by MMP2, producing a fragment containing a epidermal growth factor (EGF)-like domain, which induces integrin signaling and cell migration. These can also play a role in angiogenesis, increasing the bio-availability of vascular endothelial growth factor (VEGF), fibroblast growth factor-2 (FGF-2) and transforming growth factor-β (TGF-β), among others [26]. Ribeiro *et al.* (2010) [6] identified an increase in the activity of MMP2 and MMP1 in P-cadherin-overexpressing cells. We assessed the activity of one of these proteases, MMP2, by gelatin zymography in the conditioned media of these breast cancer cells treated with azurin and could observe a decrease in its activity.

Our data also show that the decrease in P-cadherin caused by azurin is parallel to a decrease in the phosphorylation level of FAK and Src without any alteration in total FAK and Src protein levels. It is known that FAK is necessary to the regulation of invadopodia in ovarian carcinoma cells and to promote breast cancer cell invasion [27]. Additionally, in v-Src transformed fibroblast cells, FAK promotes the formation of a v-Src-Cas-Crk-Dock180 complex, which leads to the activation of Rac1 and JNK proteins and elevated expression of MMP2 and MMP9 [28]. Also Src, when activated, can facilitate motility and invasion through reorganization of the actin cytoskeleton and disruption of normal cell-cell and cell-matrix adhesion [29]. Interestingly, it has been reported that azurin can also inhibit cancer-induced angiogenesis. Recently, p28 was reported to inhibit angiogenesis and tumor growth by inhibiting phosphorylation of VEGFR-2, FAK and Akt. These effects led to decreased motility and migration in HUVEC cells that the authors attributed to the decrease in pFAK and to a corresponding Akt-associated reduction in cell matrix attachment and survival [17]. Based on these data, we also evaluated the phosphorylation level of Akt, but did not detect any alteration in this particular protein, suggesting that a divergence occurs in the signaling pathway that regulates the invasion of breast cancer cells.

Recently, a monoclonal antibody against P-cadherin was developed by Pfizer (PF-03732010). This antibody was very effective, in anti-tumor and anti-metastatic terms, against a diverse panel of P-cadherin-overexpressing cancer models. It failed in binding to other cadherins and *in vivo* experiments showed that it is able to reduce lymph node metastases and to decrease the number of tumor circulating cells [30]. It is interesting to note that azurin displays, at least in part, some of the same effects as those mediated by a particular monoclonal antibody offering a possible new therapeutic strategy to this particular type of breast carcinomas.
